# Case report: A hybrid technique for a safe nephrectomy in a giant kidney angiomyolipoma

**DOI:** 10.3389/fsurg.2022.955932

**Published:** 2022-10-11

**Authors:** Vincenzo Vento, Andrea Benedetto Galosi, Andrea Ranghino, Luca Montecchiani, Luca Felici, Silvia Loggi, Elisabetta Cerutti, Giulio Milanese, Carmine Franzese, Daniele Castellani, Emanuele Gatta

**Affiliations:** ^1^ Vascular Surgery Department, Lancisi Cardiovascular Center, Azienda Ospedaliero-Universitaria Ospedali Riuniti di Ancona, Ancona, Italy; ^2^ Urology Unit, Azienda Ospedaliero-Universitaria Ospedali Riuniti di Ancona, Ancona, Italy; ^3^ Department of Specialist Clinical Science and Odontostomatology, Marche Polytechnic University, Ancona, Italy; ^4^ Nephrology, Dialysis and Renal Transplantation Unit, Azienda Ospedaliero-Universitaria Ospedali Riuniti di Ancona, Ancona, Italy; ^5^ Department of Intensive Care Unit, Azienda Ospedaliero-Universitaria Ospedali Riuniti di Ancona, Ancona, Italy

**Keywords:** angiomyolipoma, endovascular procedures, nephrectomy, tuberous sclerosis, rare diseases

## Abstract

**Background:**

Giant angiomyolipoma is usually associated with genetic syndromes and complications (spontaneous rupture and bleeding, hematuria, hypertension) and mass-related symptoms (flank and abdominal pain).

**Case presentation:**

We present a case of a 20-year-old woman suffering from tuberous sclerosis who was referred to our hospital with a giant angiomyolipoma causing abdominal pain. A contrast-enhanced computed tomography showed a left angiomyolipoma, measuring 28 cm × 17 cm × 27 cm. After a multidisciplinary team discussion, the patient was submitted for a nephrectomy. Percutaneous temporary occlusion of the main renal artery was achieved through an endovascular balloon catheter. Through the balloon catheter guidewire, 2,500 IU of heparin was infused to reduce the risk of tumor vein thrombosis and venous embolism. This allowed a safe kidney manipulation through a left thoracoabdominal approach. The postoperative course was uneventful. Pathology showed a 40 cm × 30 cm × 9 cm and 10 kg AML. One year after surgery, the patient is on follow-up, and her estimated glomerular filtration is 120.5 ml/min/1.73 m^2^.

**Conclusion:**

The present case showed that the endovascular control of the main renal artery could be considered a useful approach to safely managing huge renal masses when renal hilar control is expected to be very difficult.

## Introduction

Renal angiomyolipoma (AML) is a benign and uncommon solid renal tumor. Two types of AMLs are described, including sporadic (80%) and those associated with genetic syndromes (20%) such as tuberous sclerosis complex (TSC), von Hippel–Lindau syndrome, and neurofibromatosis type 1 (1, 2). Small AMLs (<4 cm) can be managed conservatively and monitored with a yearly ultrasound or computed tomography (CT) scan (2, 3), while large and growing masses, with complications (spontaneous rupture and retroperitoneal bleeding, hematuria, hypertension) or with mass-related symptoms (flank pain, abdominal pain) usually require treatments (4). Patients affected by TSC with large AMLs experience spontaneous hemorrhages in 25%–50% of cases, leading to emergency treatments that can be associated with poor outcomes (4). Therefore, elective treatments are surgical resection or selective artery embolization. In huge lesions, surgery is the treatment of choice for symptoms or complications. Surgical management of huge lesions consists of nephrectomy with early arterial ligation. However, this can be hazardous when the giant renal mass shields the aorta and vena cava entirely. In addition, AMLs are composed of large and frail networks of veins that cover the squishy tumor, and therefore, mass handling and displacement must be performed carefully to avoid bleeding (5). In some cases, it is advisable for a preoperative artery embolization to be performed several days or hours before surgery as a separate procedure (6). However, this preoperative approach has the disadvantage of involving staged procedures and higher costs. We report a case of a giant AML associated with TSC treated using a novel one-stage hybrid approach.

## Case presentation

A 20-year-old Caucasian woman was referred to our hospital with a rapidly growing abdominal mass associated with anemia and mild abdominal and left flank pain. Past medical history was positive for sporadic TSC diagnosed 9 years before. She was on mTOR inhibitor therapy for a cerebral subependymal giant cell astrocytoma. The patient reported a history of abdominal swelling in previous years and was on follow-up for bilateral angiomyolipoma since early infancy. The physical examination showed a palpable abdominal tender mass extending from the left flank to the right iliac fossa. She had no dermatologic lesions.

**Figure 1:**
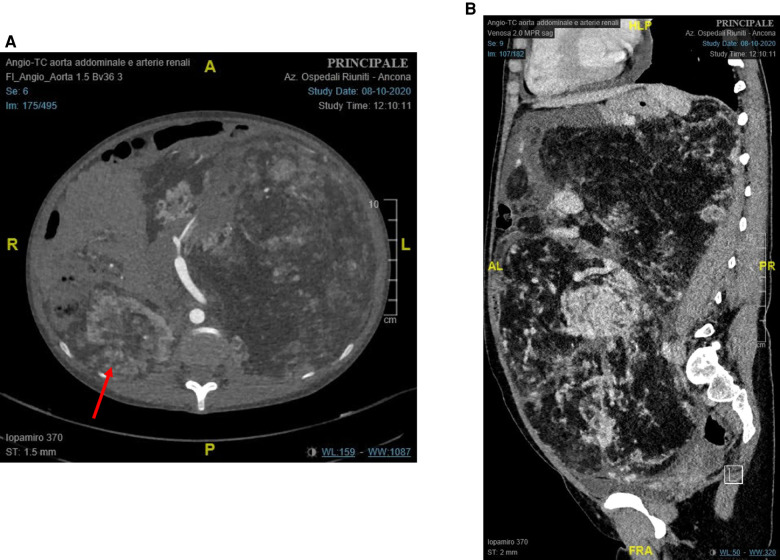
Preoperative contrast-enhanced computed tomography of the abdomen: (**A**) axial plane (red arrow: right renal angiomyolipoma); and (**B**) sagittal plane.

A contrast-enhanced CT scan showed a left renal tumor of 28 cm × 17 cm × 27 cm (Figure 1), suggestive of a giant angiomyolipoma, occupying the entire abdominal cavity and shifting abdominal organs. A 10 cm × 6.7 cm × 7.9 cm angiomyolipoma was also present in the right kidney (Figure 1A). The left renal artery and vein were dilated to 11 and 26 mm, respectively. The estimated glomerular filtration was 127 ml/min/1.73m^2^. She denied hematuria.

A careful multidisciplinary assessment involving urologists, vascular surgeons, anesthesiologists, and nephrologists was carried out. m-TOR treatment was considered ineffective, while it could be considered an option in renal cell carcinoma to shrink the tumor (7). Due to the kidney size, location, and bleeding risk, nephrectomy with a hybrid approach was suggested.

**Figure 2:**
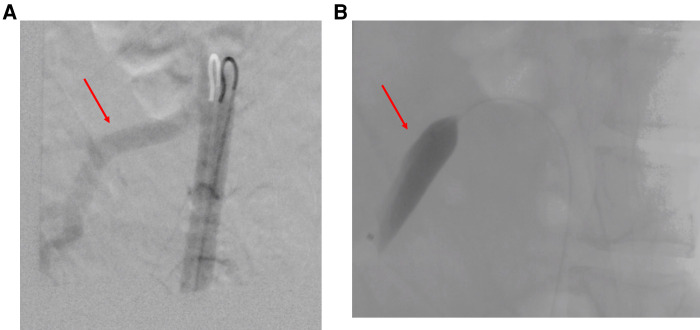
(**A**) Intraoperative left renal artery angiography (red arrow: main renal artery). (**B**) Endovascular clamping of the left renal artery (endovascular balloon occluding the main renal artery).

**Figure 3:**
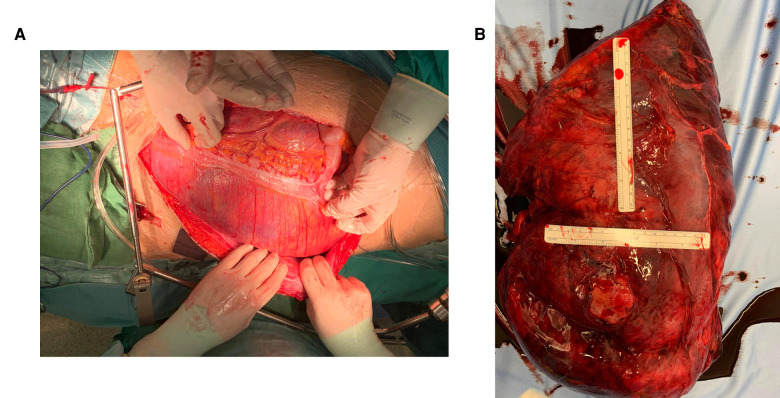
(**A**) Left thoraco-phreno-laparotomy. (**B**) Surgical specimen.

**Figure 4:**
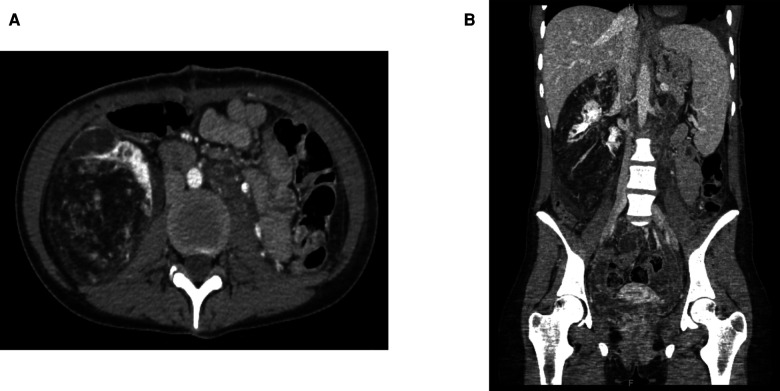
Postoperative contrast-enhanced computed tomography of the abdomen: (**A**) axial plane; (**B**) sagittal plane.

Percutaneous temporary occlusion of the main renal artery was performed in the same session of nephrectomy. Surgery began with a preliminary angiography through a left femoral artery percutaneous access to evaluate the left renal artery (Figure 2A). The renal artery was end-clamped using a 12 mm × 40 mm angioplasty balloon (Cordis Powerflex Pro, Cordis, Miami Lakes, FL, USA) (Figure 2B). Through the balloon catheter guidewire, 2,500 IU of heparin was infused to reduce the risk of tumor vein thrombosis and venous embolism. By a left thoracic-phreno-laparotomy at the level of the ninth intercostal space, a retroperitoneal kidney dissection was performed (Figure 3A). After careful isolation of the kidney, the renal artery was progressively accessed and exposed after kidney displacement. Then, the endovascular balloon was removed, and the main renal artery was ligated and sectioned. Nephrectomy was completed after isolation, ligation, and section of the renal vein. Surgical time was 234 min. The estimated blood loss was 300 ml. A left thoracic drain was left in place for 72 h. The postoperative course was uneventful, and the patient required no blood transfusion. The patient was discharged on the sixth postoperative day. A postoperative CT scan showed a complete resection (Figure 4). Pathological examination revealed a tumor measuring 40 cm × 30 cm × 9 cm and 10 kg of weight and confirmed the presence of AML (Figure 3B).

According to recent recommendations (8), the patient has started treatment with everolimus to reduce the growth of the right AML and cerebral subependymal giant cell astrocytoma. One year after surgery, the patient is still under everolimus treatment and in follow-up. Her estimated glomerular filtration is 120.5 ml/min/1.73m^2^, with no proteinuria or hematuria. The right angiomyolipoma grew up to 11.1 cm × 6.9 cm × 8.1 cm, but the patient refused partial nephrectomy and is currently asymptomatic.

## Discussion

This case report has described the largest renal AML ever reported, outlining how it was safely treated with a hybrid surgical technique. Usually, surgical treatment is indicated in AML when facing lesions over 4 cm or complications (spontaneous rupture, tumor hemorrhage, hematuria) and/or mass-related symptoms (flank pain, abdominal pain) appear (2). AMLs associated with TSC usually present with multiple, large, and symptomatic tumors. In a low percentage of large AML (3%), the smooth muscle may develop a proliferation of epithelioid cells that have a potential malignant behavior, known as epithelioid angiomyolipoma (5). It is not possible to differentiate the latter with AML by CT scanning or magnetic resonance imaging. Therefore, the progressively growing mass must be removed. However, every surgery should rely on a nephron-sparing approach whenever possible to preserve renal function (9). Conversely, nephrectomy is indicated only when a renal AML is very large, in case of suspicion of malignancy or when other treatment options cannot be performed (10). In these cases, an elective treatment is indicated. In huge AML, surgery is the treatment of choice to relieve symptoms. In large masses, artery embolization has been used several days or hours before surgery as a separate preoperative procedure (6). Indeed, one of the largest renal AMLs published in the literature was reported by Taneja et al. (39 cm × 25 cm × 9 cm, weight 7.5 kg) (11). The authors performed an open nephrectomy several days after renal artery embolization. This preoperative approach has various disadvantages in giant tumors, such as pain, hemodynamic instability, fever-induced necrosis, renal vein thrombosis, and embolism, as well as high costs related to embolization, anesthesia, and staged procedures.

The hybrid one-stage approach that we presented here was based on concomitant endovascular renal artery clamping followed by nephrectomy. This approach has two main advantages. First, surgical maneuvers to achieve renal artery ligation were performed safely, reducing the risk of massive bleeding arising from the breakdown of a large and subtle venous network surrounding the mass. The second advantage was that we controlled the hemodynamic impact of clamping on cardiac venous backflow as the AML weight was almost 18% of the patient's weight (57 kg). Furthermore, according to the anesthesiologist's monitoring feedback, artery clamping was progressively applied. Consequently, a moderate decrease in cardiac venous return was observed and managed accordingly. In addition, the risk of renal vein thrombosis and subsequent embolism was reduced by injecting heparin through the angiographic catheter.

The thoracic-phreno-laparotomy approach was chosen to allow safe manipulation and access to the left renal hilum, displacing the huge mass and making open surgery a viable option for these huge kidney tumors in the modern era of robotic-assisted surgery.

## Conclusion

In conclusion, the hybrid one-stage approach we described in this case was based on intraoperative endovascular renal artery clamping and surgical resection by the thoracoabdominal approach. This novel technique offers two main advantages: a safer approach to reaching the renal hilum and a safer way to manage the decrease of cardiac venous backflow, as well as being easy and time-saving. This technique could be useful to safely manage huge renal masses when hilar control is expected to be very difficult, such as in our case.

## Data Availability

The original contributions presented in the study are included in the article/Supplementary Material, further inquiries can be directed to the corresponding author/s.

## References

[1] Curatolo P Bombardieri R Jozwiak S. Tuberous sclerosis. Lancet. (2008) 372:657–68. 10.1016/S0140-6736(08)61279-9 18722871

[2] Restrepo JCÁ Millan DAC Sabogal CAR Bernal AFP Donoso WD. New trends and evidence for the management of renal angiomyolipoma: a comprehensive narrative review of the literature. J Kidney Cancer VHL. (2022) 9:33–41. 10.15586/jkcvhl.v9i1.177 35096516 PMC8792032

[3] Jinzaki M Silverman SG Akita H Nagashima Y Mikami S Oya M. Renal angiomyolipoma: a radiological classification and update on recent developments in diagnosis and management. Abdom Imaging. (2014) 39:588–604. 10.1007/s00261-014-0083-3 24504542 PMC4040184

[4] Steiner MS Goldman SM Fishman EK Marshall FF. The natural history of renal angiomyolipoma. J Urol. (1993) 150:1782–6. 10.1016/s0022-5347(17)35895-0 8230504

[5] Mazzucchelli R Galosi AB Scarpelli M Lopez-Beltran A Cheng L Montironi R. Contemporary update on pathology-related issues of adult renal neoplasms. Anal Quant Cytopathol Histopathol. (2014) 36:1–8. PMID: 24902365

[6] Shikino K Ikusaka M. Giant bilateral sporadic renal angiomyolipoma. CMAJ. (2016) 188:821. 10.1503/cmaj.150915 27160876 PMC4978580

[7] Galosi AB Papaveri A Castellani D Agostini E Burattini L Dell’Atti L. Level IV tumor thrombus in non-metastatic renal cell cancer? No, thanks. Level II is better. Lessons learned from a case report. Urol Case Rep. (2021) 37:101660. 10.1016/j.eucr.2021.101660 33868935 PMC8044637

[8] Curatolo P Bjørnvold M Dill PE Ferreira JC Feucht M Hertzberg C The role of mTOR inhibitors in the treatment of patients with tuberous sclerosis complex: evidence-based and expert opinions. Drugs. (2016) 76:551–65. 10.1007/s40265-016-0552-9 26927950

[9] Dell’Atti L Scarcella S Manno S Polito M Galosi AB. Approach for renal tumors with low nephrometry score through unclamped sutureless laparoscopic enucleation technique: functional and oncologic outcomes. Clin Genitourin Cancer. (2018) 16:e1251–6. 10.1016/j.clgc.2018.07.020 30122517

[10] Flum AS Hamoui N Said MA Yang XJ Casalino DD McGuire BB Update on the diagnosis and management of renal angiomyolipoma. J Urol. (2016) 195:834–46. 10.1016/j.juro.2015.07.126 26612197

[11] Taneja R Singh DV. Giant renal angiomyolipoma: unusual cause of huge abdominal mass. J Clin Imaging Sci. (2013) 3:56. 10.4103/2156-7514.122326 24404415 PMC3883275

